# Supplementary material for: Ultraviolet optical horn antennas for label-free detection of single proteins
Source: Nat Commun. 2022 Apr 5;13:1842. doi: 10.1038/s41467-022-29546-4 (PMC8983662; doi:10.1038/s41467-022-29546-4)
Supplement: Supplementary file 1 — Supplementary Information [file 41467_2022_29546_MOESM1_ESM.pdf]

Supplementary Information for

Ultraviolet optical horn antennas for label-free detection of single proteins

Aleksandr Barulin,[1] Prithu Roy,[1] Jean-Benoît Claude,[1] Jérôme Wenger[1,*]

[1] *Aix Marseille Univ, CNRS, Centrale Marseille, Institut Fresnel, 13013 Marseille, France*

** Corresponding author: jerome.wenger@fresnel.fr*

Contents:

S1. Microwave horn antenna analogy

S2. FDTD simulations of dipole emission

S3. Horn antenna fabrication

S4. FCS and lifetime data tables for p-terphenyl

S5. FCS noise analysis confirms fluorescence enhancement

S6. Additional autofluorescence time traces

S7. Background when no protein is present

S8. Comparison of single protein autofluorescence time traces with and without horn antenna

S9. Dependence with the aperture diameter

S10. Fluorescence lifetime measurements of single label-free proteins

S11. Fluorescence enhancement of diffusing β-galactosidase proteins with horn antennas

S12. β-galactosidase autofluorescence spectra in presence of urea

S13. Diffusion time of β-galactosidase and viscosity calibration in presence of urea

S1. Microwave horn antenna analogy

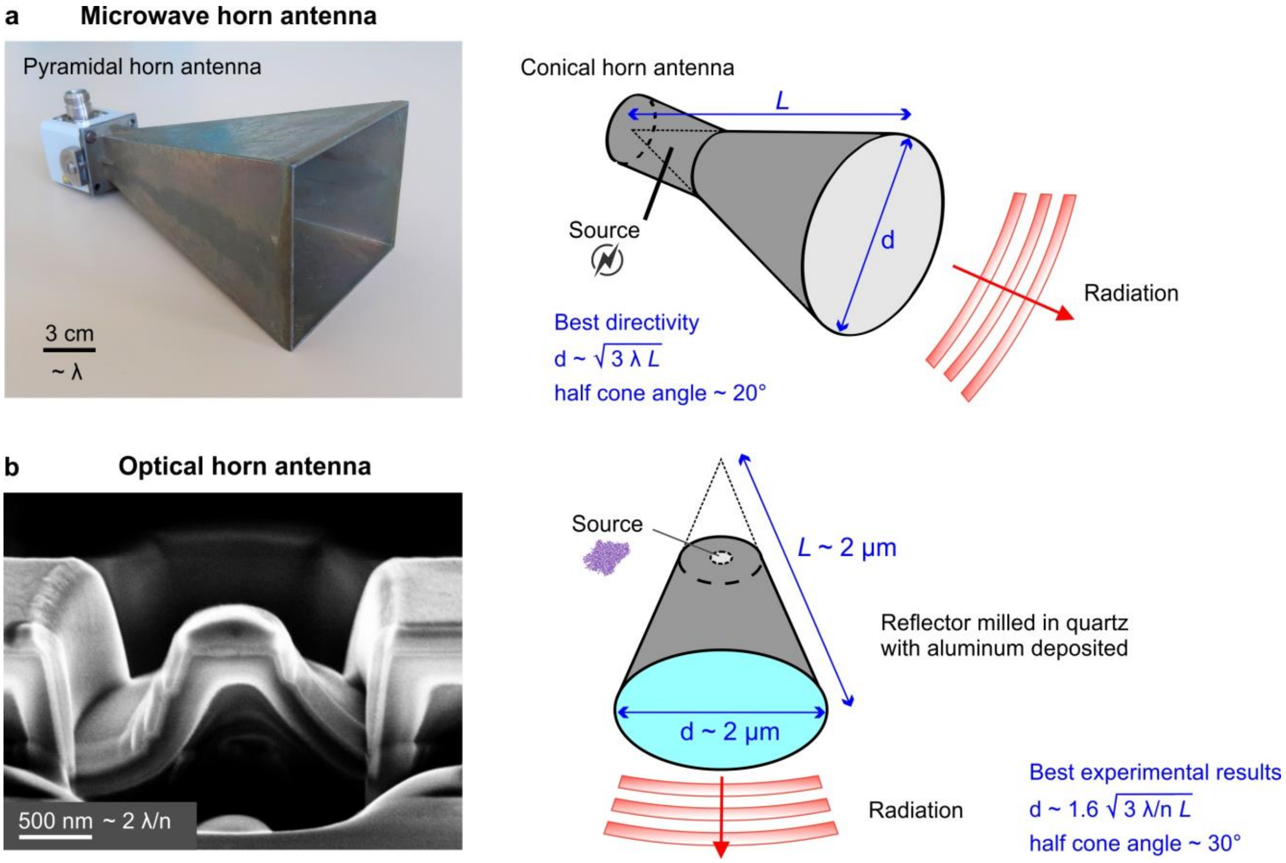

Figure S1. Analogy between microwave horn antennas (a) and optical horn antennas (b). Due to the circular symmetry of optical components (including the microscope objective), the conical horn design is most adapted. For microwave horn antennas, the directivity is optimized for a specific relationship between the antenna dimensions and the wavelength.[1] For our optical horn antennas, we retrieve a quite similar relationship, yet the large 310-410 nm spectral bandwidth in optics, the difficulty to control accurately the horn shape at the nanometer scale and the presence of resonances complicate the direct extrapolation of the microwave design formulas.[2]

S2. FDTD simulations of dipole emission

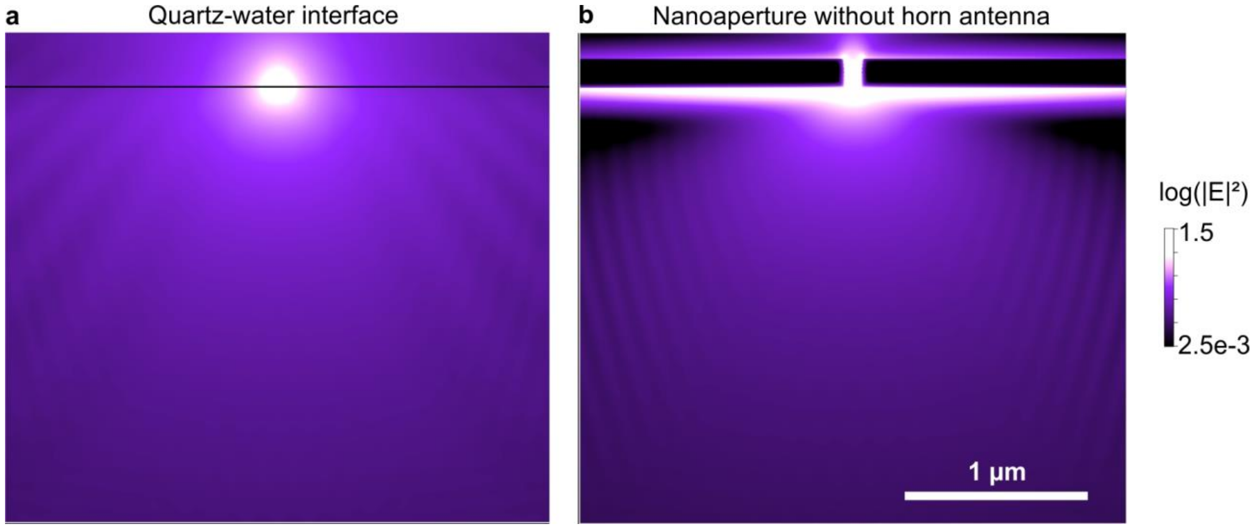

Figure S2. Finite difference time domain (FDTD) simulation of the emission pattern of a dipole located 10 nm above the quartz interface (a) and in presence of a 65 nm aluminum nanoaperture (b). The simulations are performed in the same conditions as in Fig. 1b, the colorscales are identical. In the absence of the horn antenna, the emission lies largely outside the 33° maximum collection angle of the 0.8 NA microscope objective.

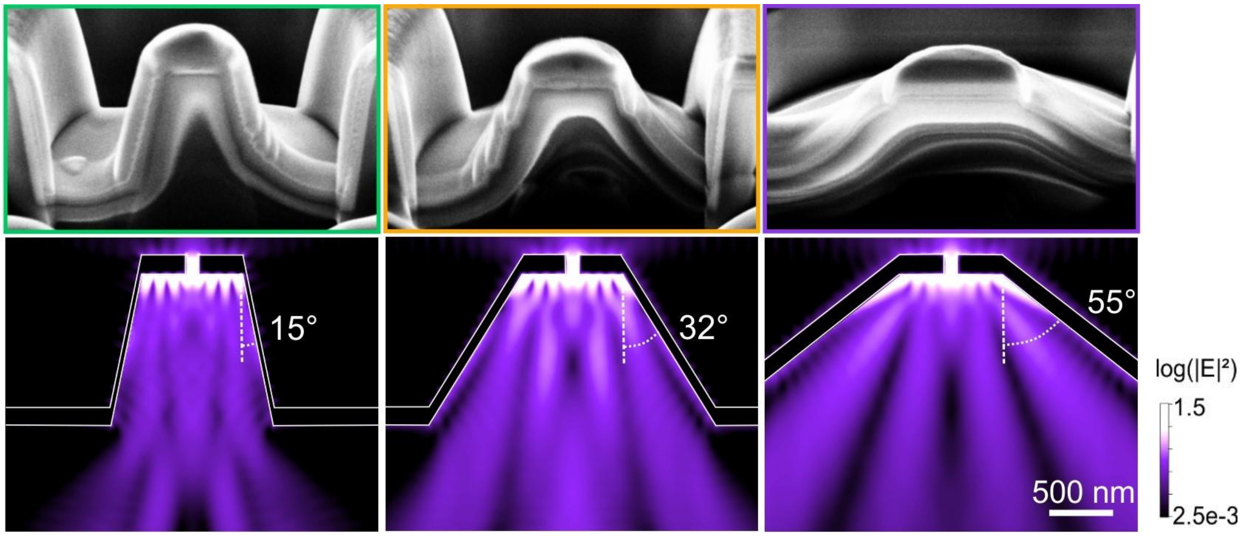

Figure S3. FDTD simulation of the emission pattern of a dipole located in the center of the 65 nm aperture, 10 nm above the quartz interface in presence of the conical horn antenna with different angles. The selected geometries reproduce the experimental horn antennas used in Fig. 1d,e. The colorscale is identical to Fig. 1b and Fig. S2. The contributions from horizontal and vertical dipole orientations are averaged.

S3. Horn antenna fabrication

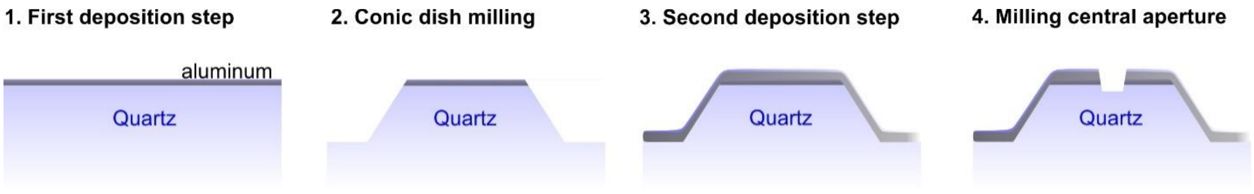

Figure S4. Fabrication protocol of the horn antenna platform: first the horn antenna is milled by focused ion beam (FIB) on a quartz coverslip coated with 50 nm aluminum layer to ensure a proper electrical conductivity (steps 1 and 2). Then a 100 nm aluminum layer is deposited on top of the horn antenna to make the horn antenna walls reflective in the UV (step 3). Finally a 65 nm diameter nanoaperture is milled in the center of the horn antenna top plateau (step 4).

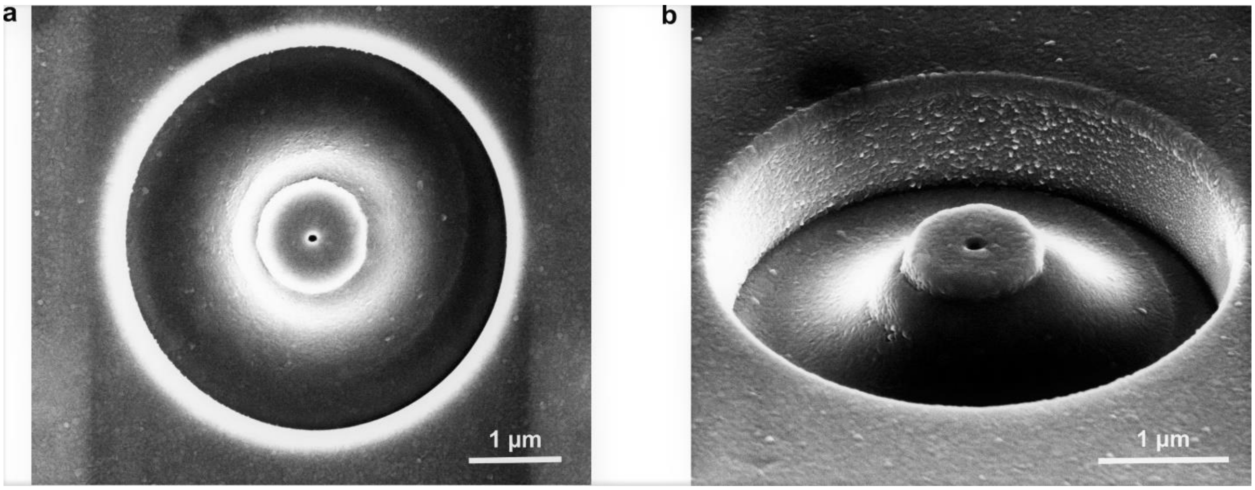

Figure S5. Scanning electron microscopy (SEM) images taken with normal incidence (a) and 52° tilt (b) of a complete horn antenna including the central 65 nm diameter nanoaperture. Similar images could be reproduced more than 10 times using the same milling parameters.

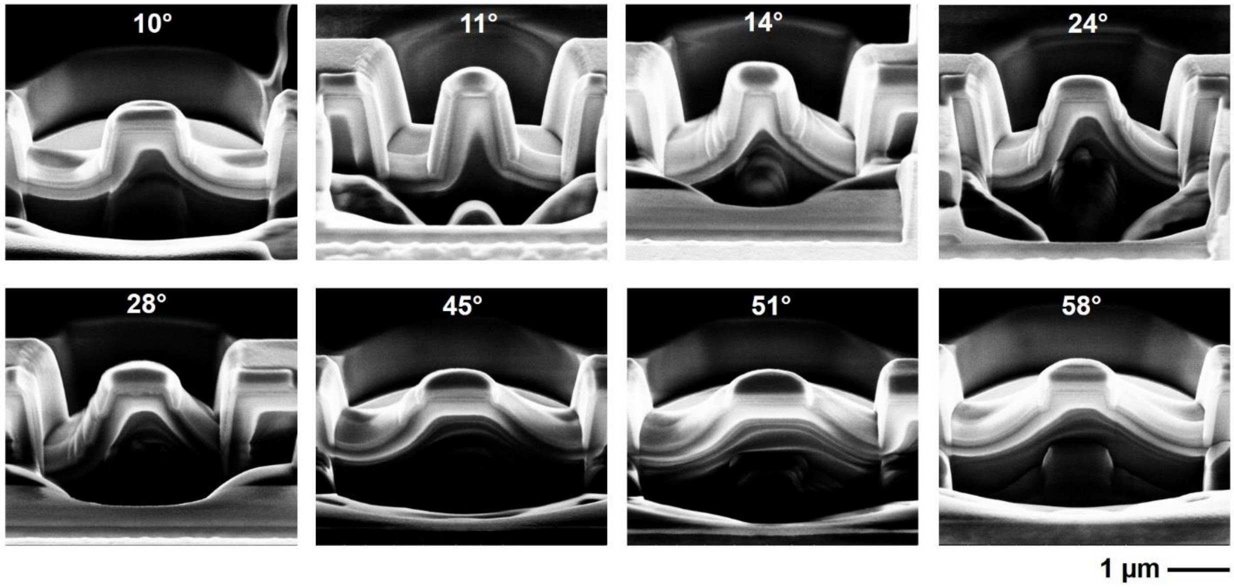

Figure S6. SEM images of different horn antennas with increasing cone angles as indicated on each picture. The sample is tilted by 52° and a FIB cross-section is performed to enable viewing the geometry of the device. The central nanoaperture has not been milled on these samples. We have checked 5 different independent samples leading to similar images.

S4. FCS and lifetime data tables for p-terphenyl

Table S1. Fit parameters for the FCS data displayed on Fig. 1d,e. We do not observe any fast blinking contribution for p-terphenyl, so n_T and τ_T are set to zero. The shape parameter κ is fixed at 8 for the confocal case based on the calibration of the microscope point spread function (PSF). For the aperture and horn antennas, we use $\kappa = 1$ based on our previous work using nanoapertures and fluorescent dyes in the visible.[3] $CRM = (F\text{-}B)/N_{mol}$ stands for the fluorescence count rate per molecule (average brightness per emitter). A similar reduction in the translational diffusion time was observed for nanoapertures in the visible spectral range as compared to the diffraction-limited confocal reference.[3]

	F **(kHz)**	***B*** **(kHz)**	N_{mol}	τ_d **(µs)**	***CRM*** **(kHz)**	**Fluo. enhancement**
Confocal	27.3	0	12.3	21.1	2.2	-
Single aperture	62.1	3.8	6.6	15.5	8.8	4
Horn antenna 32°	347.9	32	9.9	13.8	31.9	14.5
Horn antenna 15°	145	32	9.7	9.2	11.7	5.3
Horn antenna 55°	146.9	32	7.3	16.6	15.7	7.1

Table S2. Fit parameters for the fluorescence lifetime data displayed on Fig. 1f. The lifetimes are expressed in ns, the intensities are normalized so that their sum equals 1. All horn antennas and the single aperture share similar lifetime reductions. The horn antenna essentially affects the fluorescence collection, the local density of optical states is determined by the central 65 nm diameter aperture which remains constant among the different nanostructures.

	τ_1	τ_2	τ_3	I_1	I_2	I_3	**Lifetime reduction (0.95 ns / τ_2)**
Confocal	-	0.95	-	-	1	-	-
Single aperture	0.01	0.32	0.95	0.23	0.47	0.3	3
Horn antenna 32°	0.01	0.34	0.95	0.24	0.35	0.41	2.8
Horn antenna 15°	0.01	0.25	0.95	0.31	0.4	0.29	3.8
Horn antenna 55°	0.01	0.35	0.95	0.19	0.49	0.32	2.7

S5. The reduction of the FCS noise confirms the fluorescence enhancement

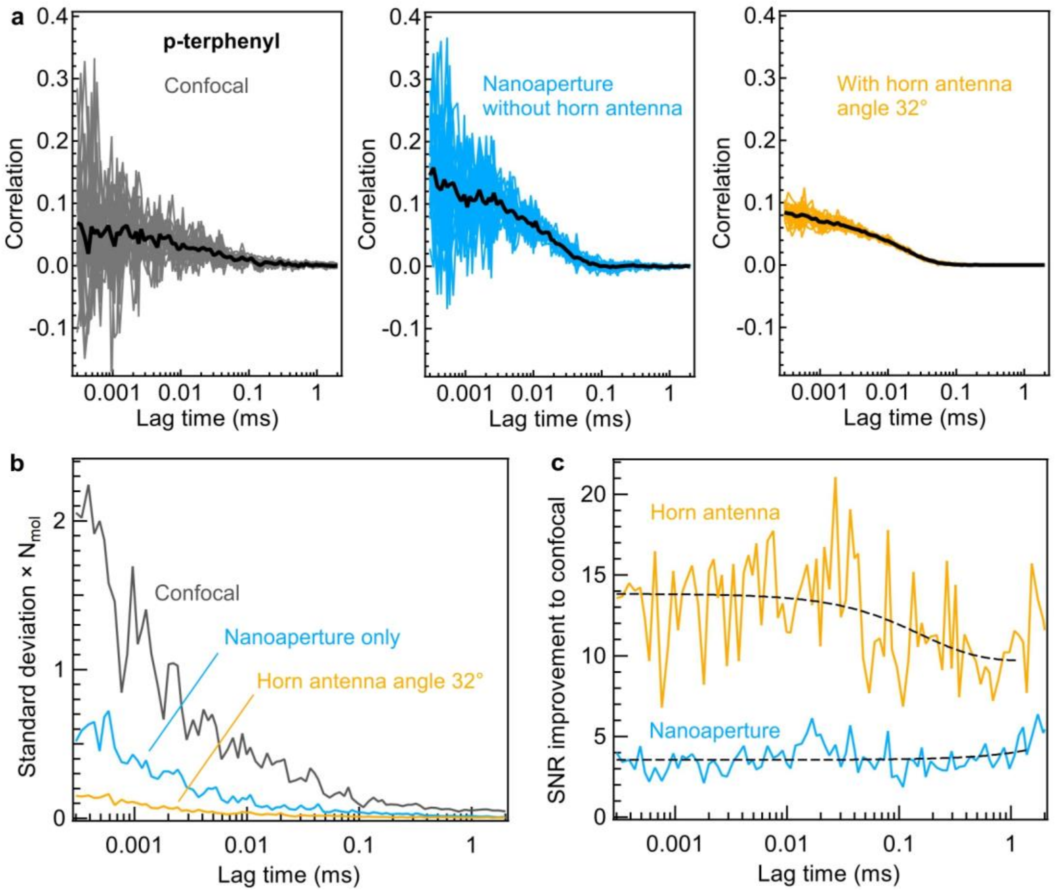

Figure S7. High fluorescence brightness per molecules improves the signal to noise ratio in FCS. (a) Raw FCS correlation functions of p-terphenyl for the confocal reference, the single 65 nm nanoaperture and the horn antenna. Each graph shows a superposition of 20 individual FCS curves (thin lines) recorded with 1 s integration time. The spread of these 1 s FCS traces represents the statistical noise of the experimental data. The thick black trace is the average data with 20 s integration time. The higher brightness obtained with the horn antenna directly translates into a reduced noise around the average value without any post-treatment analysis.[4] The concentration for the confocal data shown here is reduced by 1000x. (b) Standard deviation of the FCS trace deduced from the spread of the experimental data points in (a): for each lag time, the standard deviation is calculated among the set of FCS curves recorded with 1 s integration time. The standard deviation is then normalized by the number of molecules N_{mol} deduced from the FCS fit to obtain a concentration-independent quantity representing the noise in an FCS acquisition. These data quantify the noise reduction seen in (a) and the different cases can be directly compared. (c) The normalized standard deviation in (b) is used to compute the signal to noise ratio (SNR) improvement as compared to the confocal case. The noise in FCS depends linearly on the fluorescence brightness per molecule.[4] As this approach is only based on statistical analysis and no numerical fit is performed, this dataset independently confirms the fluorescence enhancement of the brightness per molecule deduced using FCS fitting in Fig. 1g.

S6. Additional autofluorescence decay traces

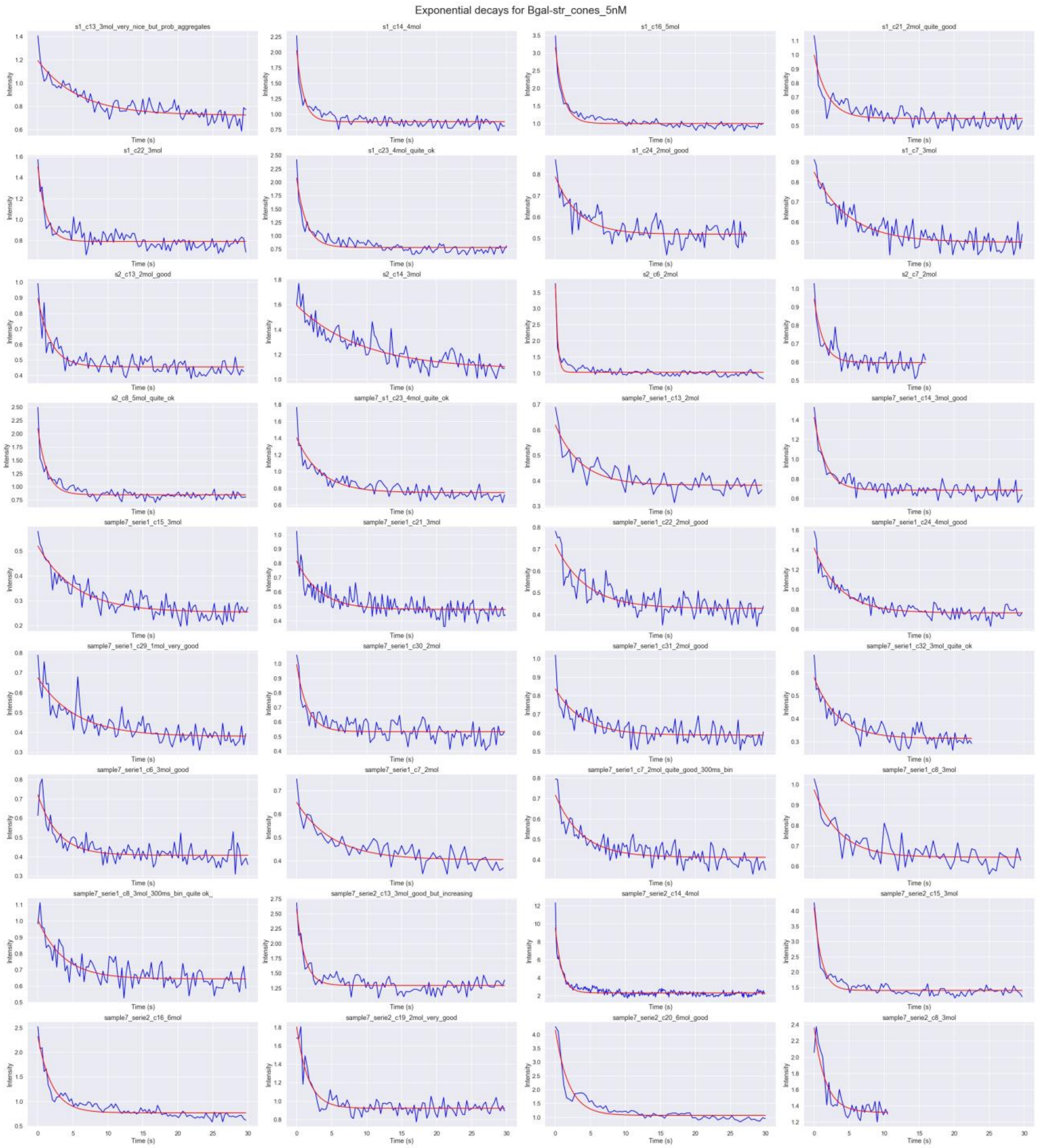

Figure S8. Autofluorescence time traces recorded on different individual horn antennas using 5 nM label-free β-galactosidase-streptavidin. The red lines are exponential fits used to extract the decay amplitude. The vertical axis is in kcounts per second.

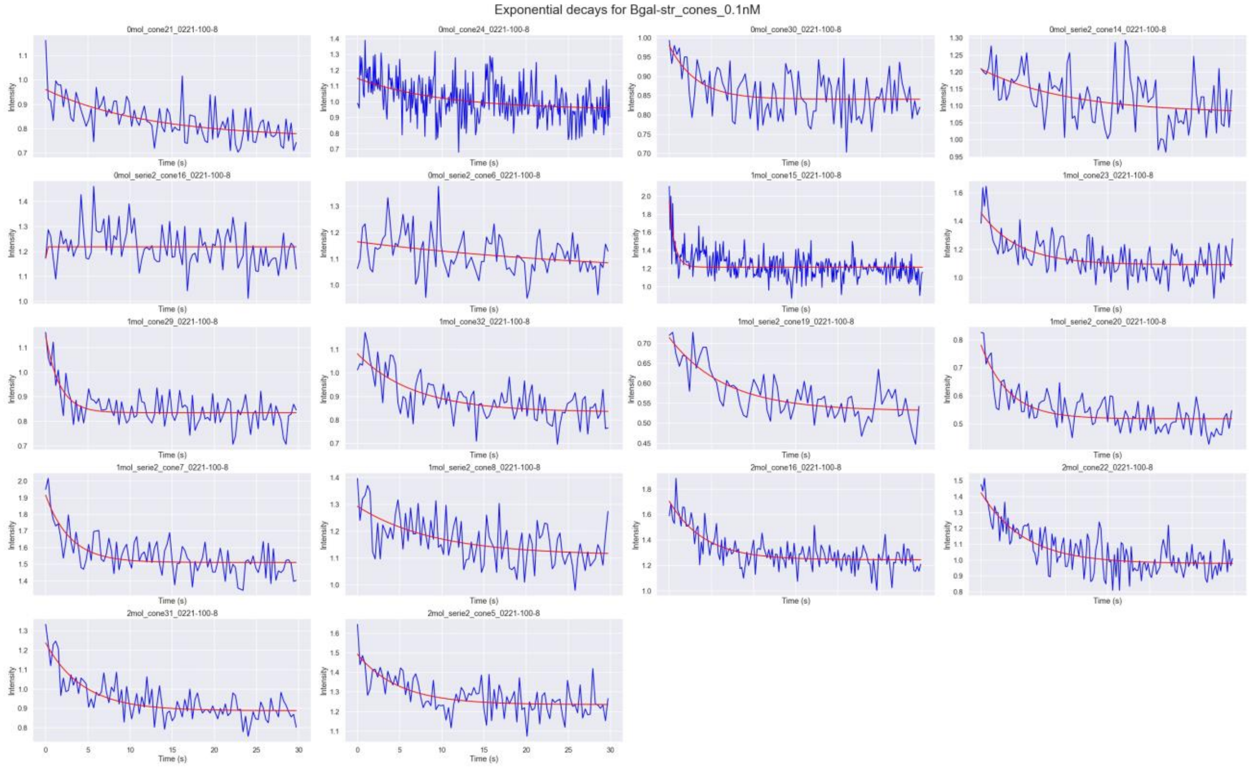

Figure S9. Autofluorescence time traces recorded on different individual horn antennas using 0.1 nM label-free β-galactosidase-streptavidin. The red lines are exponential fits used to extract the decay amplitude. The vertical axis is in kcounts per second.

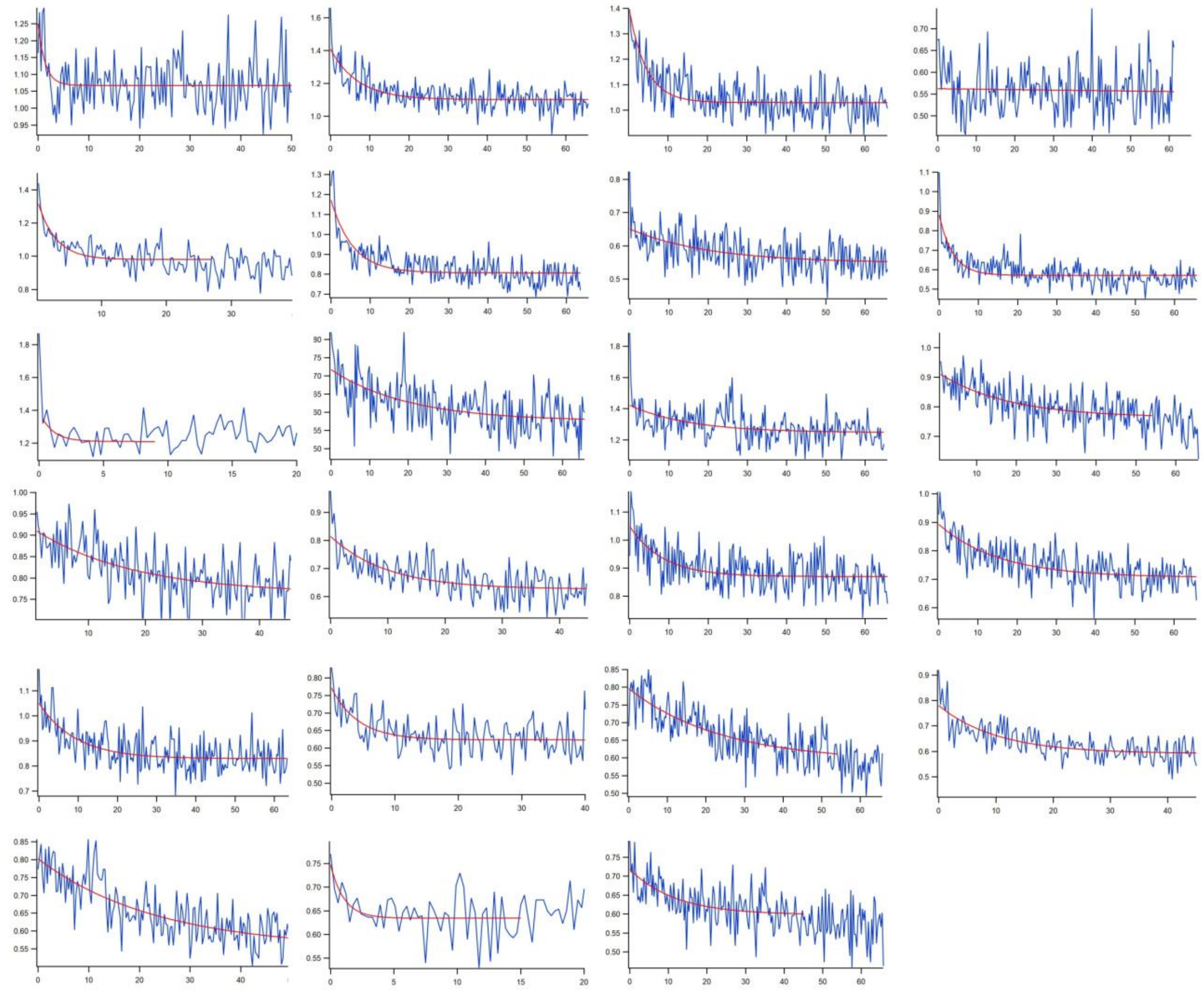

Figure S10. Autofluorescence time traces recorded on different individual horn antennas using 5 nM label-free streptavidin. The red lines are exponential fits used to extract the decay amplitude. The vertical axis is in kcounts per second, the horizontal axis is in second.

S7. Background when no protein is present

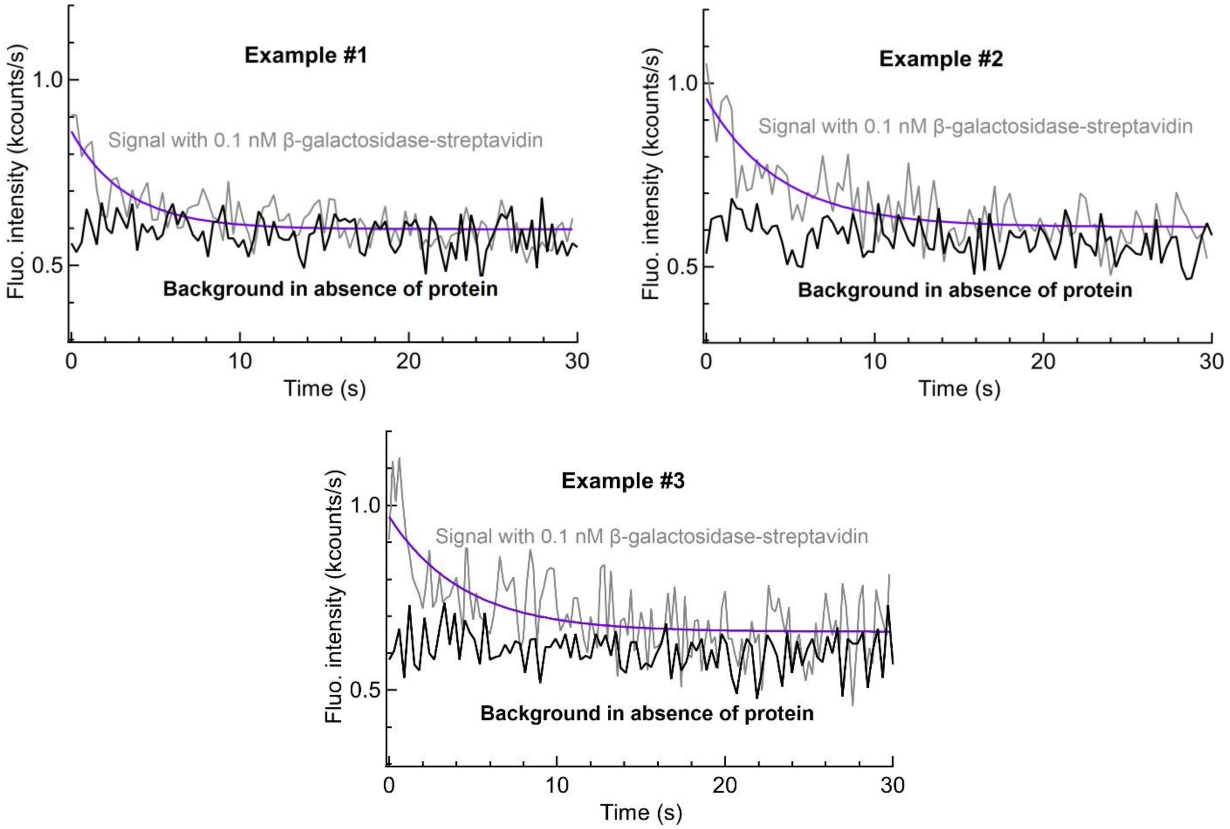

Figure S11. Autofluorescence background time traces recorded on different individual horn antennas using only the Hepes buffer solution (including the GODCAT oxygen scavenger system and 10mM DABCO). Typical time traces obtained using 0.1 nM label-free β-galactosidase-streptavidin are displayed for comparison.

S8. Comparison of single protein autofluorescence time traces with and without horn antenna

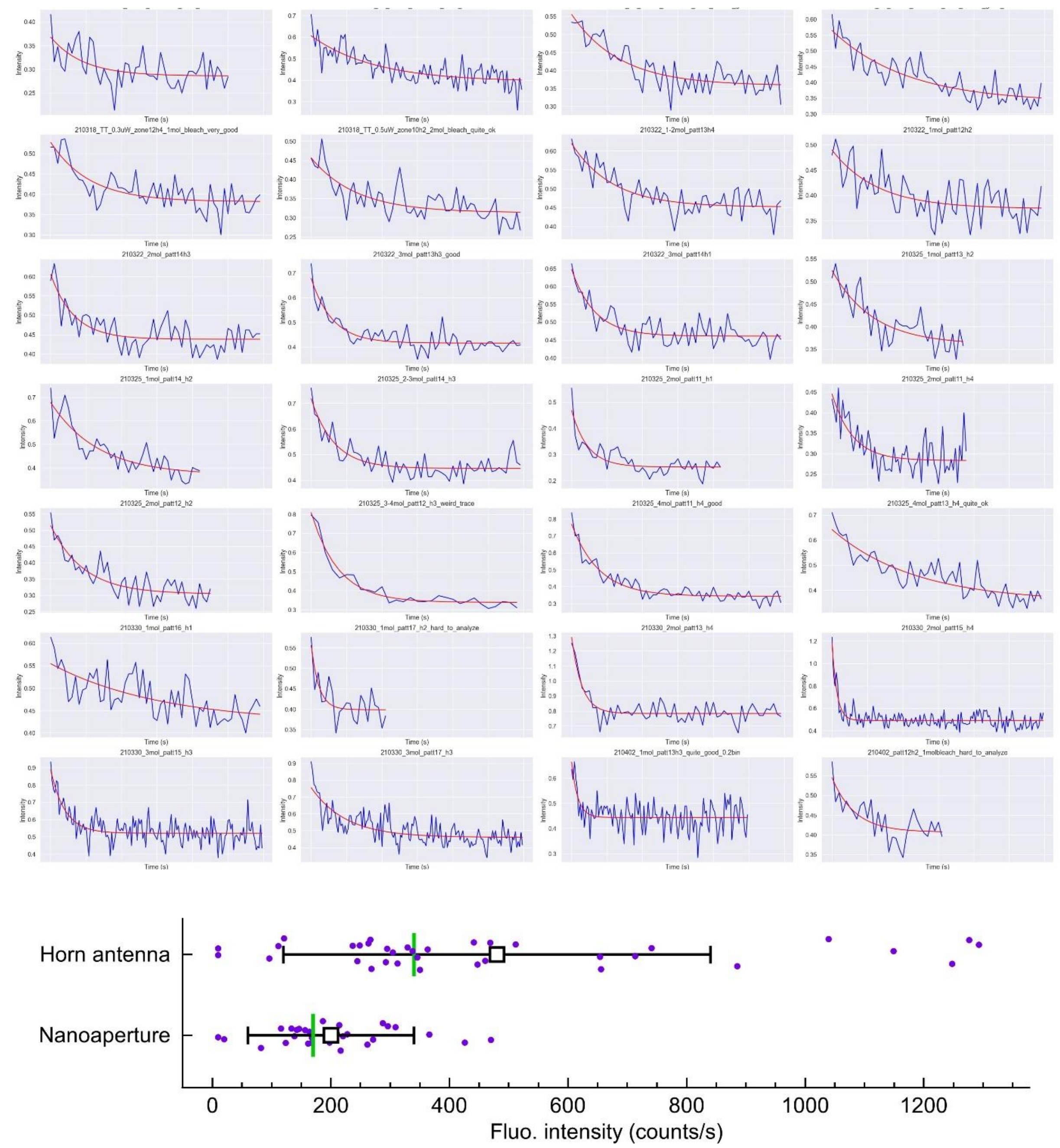

Figure S12. Autofluorescence time traces recorded on different individual nanoapertures (without conical reflector) using 5 nM label-free β-galactosidase-streptavidin. The red lines are exponential fits used to extract the decay amplitude. The vertical axis is in kcounts per second. The bottom graph compares the exponential fit amplitudes obtained from individual autofluorescence time traces for the horn antenna and the nanoaperture. The points are vertically shifted using a uniform statistical distribution. The white square marker denotes the average value with the bars extending to one standard deviation. The green vertical line indicates the median. The number of measurements are 35 with the horn antenna and 28 with the nanoaperture. These data confirm the 2 to 3× signal improvement brought by the horn antenna.

S9. Dependence with the aperture diameter

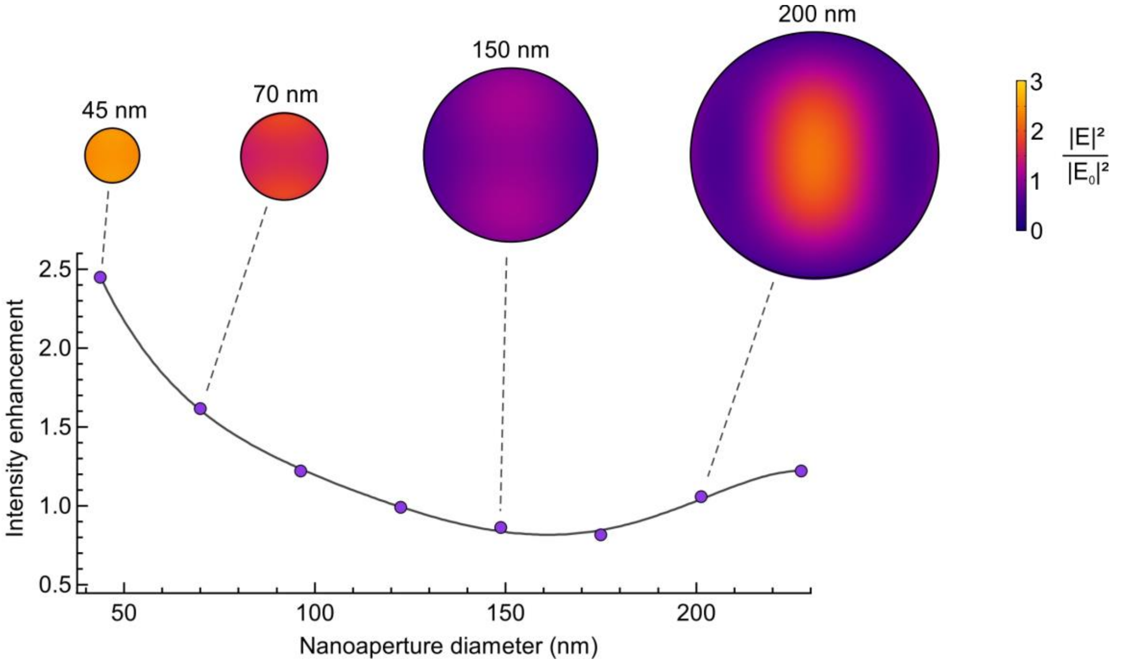

Figure S13. Numerical simulations of the 295 nm excitation intensity distributions as a function of the nanoaperture diameter. The plane of interest is taken 50 nm below the quartz-aluminum interface to reproduce the conditions used to detect proteins immobilized at the surface of the 50 nm undercut into the quartz substrate [6,7] (Fig. 2). For clarity only the aperture surface accessible to the proteins is shown here, we do not plot the field distribution into the substrate. The intensity enhancement respective to the incoming intensity is averaged over the whole aperture surface and plotted as a function of the aperture diameter. While smaller diameters feature higher local excitation enhancements, they also induce stronger quenching losses,[8,9] so the optimum diameter to maximize the net signal enhancement is shifted towards slightly larger diameters.[10] The 65 and 200 nm diameters chosen here are based on an experimental screen of the diameter influence and optimization of the signal-to-noise ratio. The grey line is a polynomial interpolation between the simulated data points.

S10. Proof-of-principle fluorescence lifetime measurements from a single label-free protein

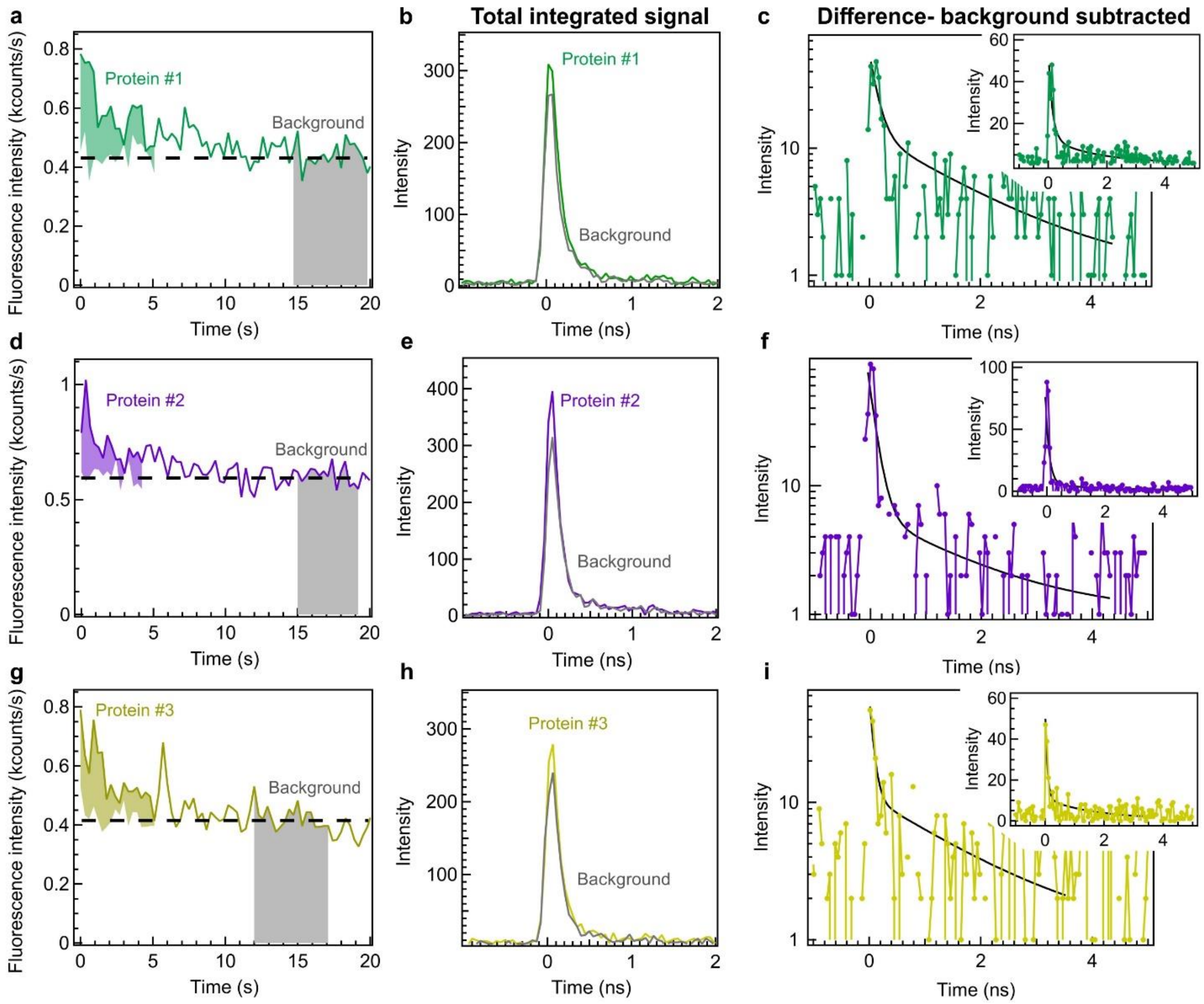

Figure S14. Single photon counting histograms from a single protein. We use here the data collected for immobilized β-galactosidase-streptavidin with 0.1 nM concentration. The traces are selected so that the decay amplitude is around 200 to 300 counts per second, which is the most representative of a single protein bleaching (Fig. 2). (a,d,g) Fluorescence time traces used for the analysis. For each selected time interval (shaded areas), the photon arrival times are sorted to compute the fluorescence lifetime decay histograms (b,e,h). The background influence is taken into account by subtracting the histogram taken once the protein of interest has bleached using the same integration time as the protein emission duration (grey traces in a,d,g). After background subtraction (c,f,i), the histogram contains the information representative of the UV photons emitted by a single protein. The histogram binning time is 48 ps. The insets in (c,fi) show the histograms on a linear vertical scale. The fit results are detailed in Tab. S3.

Measuring the fluorescence lifetime from a single molecule remains challenging, even with bright fluorescent dyes in the visible, as the limited photon budget before photobleaching is split into the different histogram channels. The data shown here demonstrates that despite the technical challenges, the high collection efficiency brought by the UV horn antenna enables extracting the lifetime histogram from a single label-free protein.

Table S3. Fit parameters for the single molecule fluorescence lifetime data in Fig. S14c,f,i. Here the 10 ps background contribution has been removed by the subtraction treatment. However, the data acquired while averaging at high µM concentration (Fig. S15b) shows that the lifetime decays of β-galactosidase are not a single exponential, but rather a bi-exponential with a long and a short component (the decays for β-galactosidase and β-galactosidase-streptavidin are similar). This multi-exponential response is typical of protein autofluorescence.[11] Owing to the limited total photon budget from a single protein, the statistical noise is large for the component with the longest lifetime. In the lifetime analysis, we have decided to fix the long lifetime component to its 1.6 ns value obtained from the averaging at 1.7 µM (Fig. S15b, Tab. S5). The lifetimes are expressed in ns, the intensities are normalized so that their sum equals 1. τ_{1-2}^{av} denotes the intensity-averaged lifetime. The instrument response function is 120 ps (full width at half-maximum) taking into account the 48 ps binning time for the lifetime histogram.

	τ_1	τ_2 *(fixed)*	I_1	I_2	τ_{1-2}^{av}
Protein #1	0.14	1.6	0.78	0.22	0.5 ± 0.3
Protein #2	0.13	1.6	0.90	0.10	0.3 ± 0.2
Protein #3	0.07	1.6	0.83	0.17	0.3 ± 0.2

S11. Fluorescence enhancement of diffusing β-galactosidase proteins with horn antennas

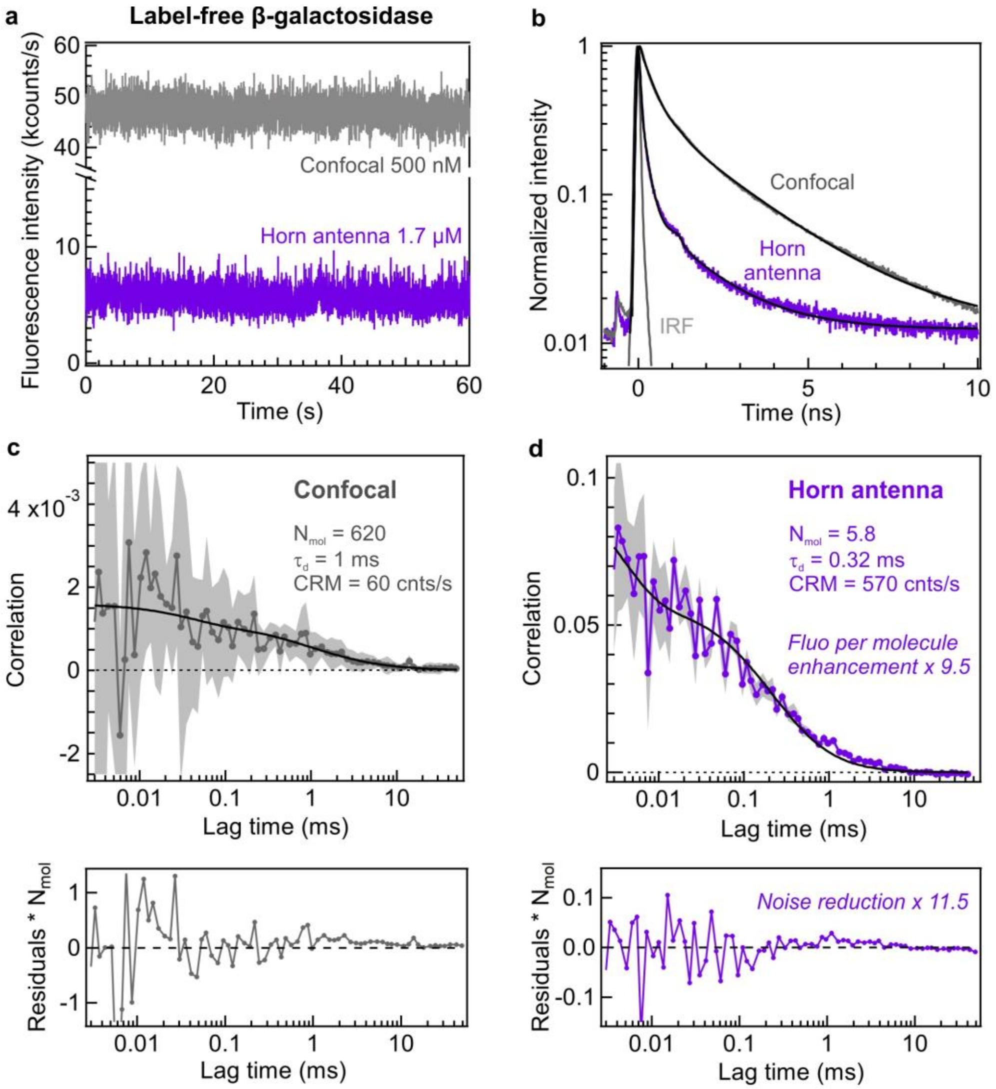

Figure S15. Demonstration of fluorescence enhancement for label-free β-galactosidase proteins inside a horn antenna with 32° cone angle and 65 nm aperture diameter. (a) Fluorescence time trace at 10 µW laser power with 10 ms bin time. The background for the confocal is 8 kHz and 2.4 kHz for the horn antenna. The background for the confocal experiment stems mostly from the UV fluorescence of the GODCAT oxygen scavenger in the 2 fL confocal volume. For the horn antenna, the GODCAT background is reduced as the detection volume is ~400× smaller, but an additional contribution comes from the photoluminescence of the metal. (b) Fluorescence lifetime decay and numerical fits. IRF is our instrument response function (full width at half maximum 160 ps). The fit results are summarized in Tab. S5. (c,d) FCS correlation functions (dots) and their numerical fits (black curves) corresponding to the time traces in (a). The grey shaded traces indicate the noise of the FCS data. The FCS fit results are summarized in Tab. S4 and the main quantities are indicated on the graph. The lower traces show the residuals from the fit functions multiplied by the number of detected molecules (inverse of correlation amplitude). These

normalized residuals do not depend on the protein concentration so that their amplitudes can be directly compared. The acquisition times are identical here with 130 s. To quantify the reduction of the normalized residuals, we sum their absolute values in the 10 µs - 1 ms lag time interval. We observe a 9.5 ± 2.0 × increase of the CRM brightness per molecule. This fluorescence enhancement is supported by the 11.5× reduction of the normalized residuals amplitude as the noise in FCS scales directly proportional to the brightness per emitter.[4] As a side note, our data indicates that a fast blinking contribution (n_T, τ_T) is needed to yield flat residuals. The precision is limited for these two parameters, but they do not have any influence on our main conclusions. Different processes could explain this contribution in the sub-10 µs range such as dark state blinking, fast protein conformation changes, metal quenching in close nanometer proximity to the surface, and/or residual contribution from the photodetector afterpulsing. Currently we cannot distinguish between these potential sources, but the photokinetics in the sub-10 µs range do not impact any of our main conclusions.

Table S4. Fit parameters for the FCS data displayed on Fig. S15c,d. The shape parameter κ is fixed at 8 for the confocal case and 1 for the aperture and horn antennas. The 295 nm laser power is 10 µW. The β-galactosidase concentration is 500 nM for the confocal case and 1.7 µM for the horn antenna.

	F **(kHz)**	***B*** **(kHz)**	N_{mol}	n_T	τ_T **(µs)**	τ_d **(ms)**	***CRM*** **(Hz)**	**Fluo. enhancement**
Confocal	47.1	8	620	0.4	40	1	60 ± 10	-
Horn antenna 32°	5.7	2.4	5.8	0.75	4	0.32	570 ± 30	9.5 ± 2.0

Table S5. Fit parameters for the fluorescence lifetime data in Fig. S15b. The lifetimes are expressed in ns, the intensities are normalized so that their sum equals 1. τ_{2-3}^{av} denotes the intensity-averaged lifetime of the 2nd and 3rd components (discarding the 10 ps background contribution) and is used as a read-out lifetime to demonstrate the lifetime reduction with the optical horn antenna.

	τ_1	τ_2	τ_3	I_1	I_2	I_3	τ_{2-3}^{av}
Confocal	0.01	0.39	2.38	0.07	0.24	0.69	1.9 ± 0.1
Horn antenna 32°	0.01	0.20	1.61	0.48	0.20	0.32	1.1 ± 0.1

S12. β-galactosidase autofluorescence spectra in presence of urea

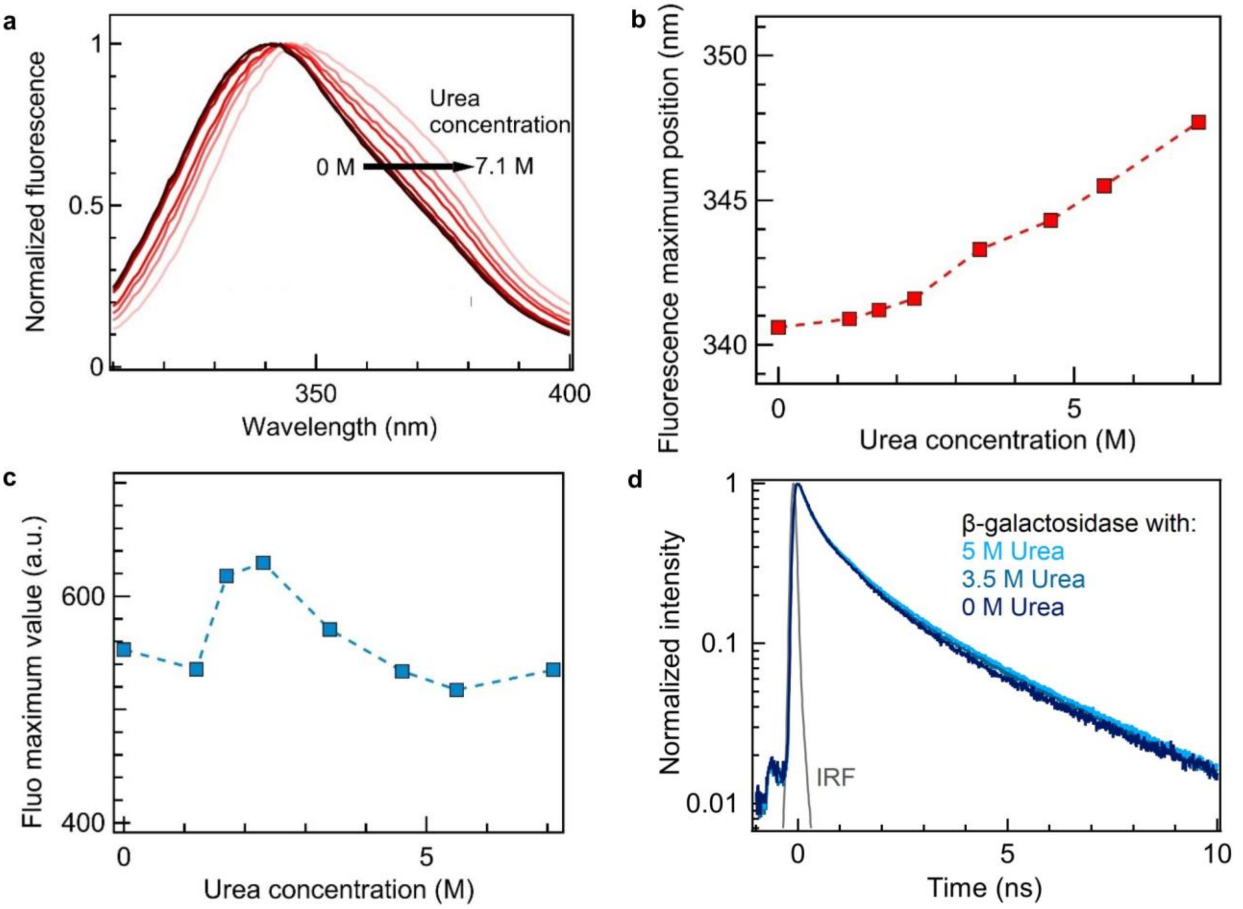

Figure S16. (a) Autofluorescence spectra of β-galactosidase for increasing concentrations of urea. Upon unfolding and denaturation, the autofluorescence spectra maximum shifts towards larger wavelengths (b), while the maximum intensity at the fluorescence peak stays rather similar (c). These spectra were recorded on a Tecan Spark 10M spectrofluorometer with excitation fixed at 260 nm. The β-galactosidase concentration is 700 nM in a 300 mM NaCl, 25 mM Hepes, 0.5 % Tween20, pH 7 buffer solution. (d) Normalized fluorescence lifetime decay traces for β-galactosidase with increasing urea concentrations. No major change is detected on the fluorescence decay, the fluorescence lifetime increases by a few percent in presence of 5 M urea.

S13. Diffusion time of β-galactosidase and viscosity calibration in presence of urea

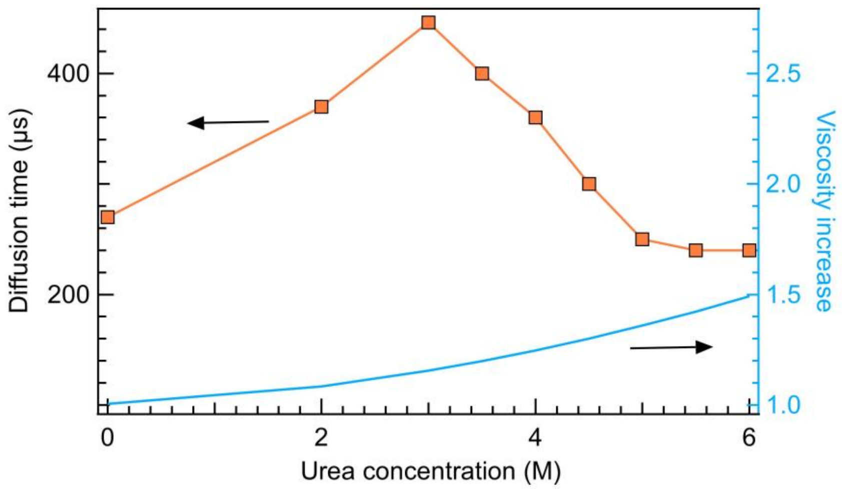

Figure S17. FCS diffusion time of β-galactosidase recorded on the horn antenna in presence of urea (left axis). This data is used to compute the hydrodynamic radius of the protein upon unfolding and denaturation shown in Fig. 4b following the approach described in [10]. As the hydrodynamic radius also depends on the viscosity of the solution, we have calibrated the viscosity increase due to the addition of urea (blue trace, right axis). This calibration is performed separately using FCS on a visible-light confocal microscope with Alexa 647 fluorescent molecules.[3]

Supplementary references

1. Balanis, C. A. *Antenna theory: analysis and design*. (John Wiley & Sons, 2005).
2. Novotny, L. & Hulst, N. van. Antennas for light. *Nature Photonics* **5**, 83–90 (2011).
3. Baibakov, M. *et al.* Extending Single-Molecule Förster Resonance Energy Transfer (FRET) Range beyond 10 Nanometers in Zero-Mode Waveguides. *ACS Nano* **13**, 8469–8480 (2019).
4. Wenger, J. *et al.* Nanoaperture-Enhanced Signal-to-Noise Ratio in Fluorescence Correlation Spectroscopy. *Anal. Chem.* **81**, 834–839 (2009).
5. Loeff, L., Kerssemakers, J. W. J., Joo, C. & Dekker, C. AutoStepfinder: A fast and automated step detection method for single-molecule analysis. *Patterns* **2**, 100256 (2021).
6. Tanii, T. *et al.* Improving zero-mode waveguide structure for enhancing signal-to-noise ratio of real-time single-molecule fluorescence imaging: A computational study. *Phys. Rev. E* **88**, 012727 (2013).
7. Wu, M. *et al.* Fluorescence enhancement in an over-etched gold zero-mode waveguide. *Opt. Express* **27**, 19002–19018 (2019).
8. Jiao, X., Peterson, E. M., Harris, J. M. & Blair, S. UV Fluorescence Lifetime Modification by Aluminum Nanoapertures. *ACS Photonics* **1**, 1270–1277 (2014).
9. Jiao, X., Wang, Y. & Blair, S. UV fluorescence enhancement by Al and Mg nanoapertures. *J. Phys. D: Appl. Phys.* **48**, 184007 (2015).
10. Barulin, A., Claude, J.-B., Patra, S., Bonod, N. & Wenger, J. Deep Ultraviolet Plasmonic Enhancement of Single Protein Autofluorescence in Zero-Mode Waveguides. *Nano Lett.* **19**, 7434–7442 (2019).
11. Lakowicz, J. R. *Principles of Fluorescence Spectroscopy*. (Springer US, 2006).
